# Early Integration of CAR T‐Cell Therapy in Mantle Cell Lymphoma: Expert Opinion in the Era of BTK Inhibitors

**DOI:** 10.1002/hon.70265

**Published:** 2026-10-08

**Authors:** Carlo Visco, Stefania Bramanti, Paolo Corradini, Enrico Derenzini, Pier Luigi Zinzani

**Affiliations:** ^1^ Department of Engineering for Innovative Medicine Hematology and Bone Marrow Transplant Unit Section of Biomedicine of Innovation University of Verona Verona Italy; ^2^ IRCCS Istituto Clinico Humanitas Rozzano Rozzano Italy; ^3^ Divisione di Ematologia Fondazione IRCCS Istituto Nazionale dei Tumori di Milano Milano Italy; ^4^ Università degli Studi di Milano Milano Italy; ^5^ Oncohematology Division IEO European Institute of Oncology IRCCS Milan Italy; ^6^ Department of Health Sciences University of Milan Milan Italy; ^7^ Dipartimento di Scienze Mediche e Chirurgiche Università di Bologna Bologna Italy; ^8^ IRCCS Azienda Ospedaliero‐Universitaria di Bologna, Istituto di Ematologia “Seràgnoli” Bologna Italy

**Keywords:** btk inhibitors, car t cells, hematology, mantle cell lymphoma, second line treatment, sequence optimization, sequence planning

## 1 Introduction

The therapeutic landscape of mantle cell lymphoma (MCL) is currently undergoing a deep transformation. The introduction of Bruton tyrosine kinase inhibitors (BTKis) in the frontline treatment, is reshaping treatment algorithms, and redefining the sequencing of available options [1, 2, 3, 4]. This evolving scenario is generating a growing unmet clinical need in the second‐line setting, for patients who relapse after exposure to BTKi. These patients often present with biologically aggressive disease and limited sensitivity to conventional salvage therapies.

CD19‐directed CAR T‐cell therapy (CAR‐T, brexucabtagene autoleucel [brexu‐cel] and lisocabtagene maraleucel [liso‐cel]) have demonstrated high response rates and durable remissions. The cohort 1 of the ZUMA 2 trial included patients previously exposed to BTKi, and lead to the approval of brexu‐cel from third line in Europe [5]. After 5 years of median follow‐up, 35% of brexu‐cel–treated patients in cohort 1 were still alive, median duration of response (DoR) was 36.5 months, and median overall survival (mOS) of 46.5 months [6]. Patients achieving complete response (CR, *n* = 46) experienced the greatest benefit with a median DoR of 46.7 months and mOS of 60.2 months, indicating that brexu‐cel can induce long‐term remissions in MCL, even when earlier lines were excluded [6]. Curiously, in the BTKi naïve cohort (#3), response rates were similar to those in cohort 1 [7].

Liso‐cel demonstrated durable responses with manageable safety in the relapsed/refractory MCL cohort of TRANSCEND NHL 001 (NCT02631044) and first real‐world data from the CIBMTR seem to confirm these results [8, 9]. Nevertheless, liso‐cel is not yet approved for reimbursment for mantle‐cell lymphoma by AIFA and real‐world data from CIBMTR have at the moment a limited follow‐up, while brexu‐cel is already approved by FDA in second line. Of note, earlier integration of CAR‐T would likely be relevant, especially for younger patients experiencing early relapse after induction therapies [9, 10, 11, 12]. Furthermore, when CAR‐T is offered in earlier line, patients may still retain more favorable physical fitness, and less aggressive clinical and biological characteristics [13].

Despite growing interest in this approach, current literature on the use of CAR‐T cells is mainly focused on outcomes in heavily pretreated populations, and a significant knowledge gap remains in this setting.

Our goals would be to produce valuable scientific content in support of clinical practice, and favor an earlier positioning of CAR‐T in Countries where this option is available only from later lines. With the approval of BTKi in the upfront treatment, a shift in the MCL therapeutic algorithm has occurred.

Therefore, there is a need for:

- Redefining therapeutic sequences in light of this shift;
- Developing early monitoring strategies for patients treated with BTKi, in order to limit the risk of drop‐off or ineligibility to CAR‐T;
- Timely patient selection to guide therapeutic decisions; Summing up, this expert opinion aims to provide concrete and up‐to‐date clinical guidance, facilitate patient monitoring, integrate relevant clinical and biological parameters (including molecular response and *TP53* mutations), and finally support the clinician in defining the individual treatment journey.

## 2 The Treatment Scenario

The treatment paradigm of relapsed/refractory patients frames post‐covalent BTKi (cBTKi) relapse as a distinct high‐risk therapeutic space, in which historical expectations of durable disease control no longer apply. CAR‐T, clinical trial participation, and novel targeted agents are prioritized, whenever possible by all available clinical guidelines [13, 14]. The interest in non‐covalent BTKis inhibitors, bispecific antibodies, antibody‐drug conjugates, and CAR‐T is rapidly growing in this scenario [15]. Importantly, current practice has largely moved away from allogeneic transplantation (allo‐SCT) in favor of CAR‐T in eligible patients [15].

Real‐world outcomes following the initiation of cBTKi as second line therapy are not as good as in clinical trials. A significant proportion of patients do not respond or have rapid progression, dose‐limiting toxicities occur, reducing de‐facto the space for CAR‐T indication, especially for patients with more aggressive disease, that are those that more benefit of this therapy [16, 17].

## 3 CAR‐T Cell Therapy

The efficacy of CAR‐T in MCL appears consistently robust across clinical trials, real‐world cohorts, reviews and pooled analyses [18]. Wan et al. also confirmed activity in biologically adverse subgroups, including *TP53*‐aberrant and blastoid disease. Pooled data support CAR‐T as the approach most likely to induce deep and prolonged remission in cBTKi pretreated MCL [18].

The US Lymphoma CAR T Consortium established that brexu‐cel was effective in standard‐of‐care practice, and subsequent registry‐based studies from Europe and North America have broadly confirmed that real‐world outcomes are comparable to those observed in pivotal trials [19]. In the Germany/Switzerland registry, CR was observed in 61% of evaluable patients, with a median DoR of 31 months, median event‐free survival of 25 months, and mOS of 41 months, despite a population enriched for *TP53* mutations, blastoid histology, and early progression [20]. Similarly, recent CIBMTR and other Consortium indicate that the benefit of brexu‐cel and liso‐cel extend beyond tightly selected trial populations and may also be meaningful in patients treated after few prior lines of therapy [8].

A recent analysis examined real‐world eligibility, use, and barriers to CAR‐T in relapsed/refractory MCL. Selection bias provided by studies that primarily reported outcomes in selected groups occurred, while the wider post‐BTKi population would include many individuals who will never proceed to infusion due to disease progression, logistical challenges, or other issues. These findings highlight a clinically significant denominator issue: the favorable outcomes observed after infusion may overstate the real‐world impact of a CAR T‐based approach when considered across the entire relapsed population [11]. This is especially important in the context of earlier‐line use, where timing, referral processes, and the ability to safely bridge patients play a crucial role in determining overall benefit. Unfortunately, most studies and trials did not include second‐line patients or did not analyze them separately [10]. However, Santoro et al. reported that brexu‐cel was associated with high remission rates and meaningful survival in patients aged > 70 years (36% infused in second line). Functional status, rather than age, would be the major factor to be considered to guide eligibility [11].

The TARMAC study tested not only CAR‐T efficacy but also a practical sequencing concept [21]. In this phase II study, ibrutinib was started before leukapheresis and continued during manufacturing and for at least 6 months after infusion in MCL; the report was explicitly motivated by preclinical and clinical evidence suggesting that ibrutinib may improve apheresis product fitness, CAR‐T expansion, and toxicity. This study therefore supports the biological plausibility of combining BTKi with CAR T‐based treatment pathways rather than treating them as mutually exclusive alternatives [21]. This combination or sequential perspective is also concordant with prior mechanistic and translational work, suggesting that BTK inhibition may reduce T‐cell exhaustion and improve CAR T‐cell function. The available data increasingly suggest that at least some MCL patients may benefit from BTKi‐supported CAR‐T strategies. In practical terms, this means that the question is no longer only whether CAR‐T should be used, but whether pre‐infusion disease control and immune optimization can improve the probability of successful delivery and durable remission [21].

Recently, a single‐institution, single‐arm pilot (*n* = 20) trial evaluating acalabrutinib plus rituximab followed by brexu‐cel in previously untreated high‐risk MCL patients showed That CAR‐T therapy is highly effective with induction of high rates of undetectable MRD state (NCT05495464, the window‐3 study) [22].

## 4 Approved Alternatives to CAR‐T

Non‐CAR T approaches are associated with a higher use of subsequent lines of therapy after the index treatment, along with shorter time to next treatment and shorter treatment‐free intervals. This pattern suggests more frequent and earlier disease progression as patients move through successive non‐CAR‐T regimens. Standardized costs were also greater during post‐index non‐CAR‐T periods compared with those following CAR‐T. These findings indicate that earlier use of CAR‐T may help limit the need to cycle through progressively more costly and less effective non‐CAR‐T treatments, potentially reducing healthcare resource utilization and the economic burden on both patients with relapsed/refractory MCL and the healthcare system.

Historically, allo‐SCT represents the only treatment with curative potential for MCL. However, despite its proven efficacy, its widespread use has been constrained by toxicity and limitations related to patient characteristics. As a result, allo‐SCT has remained a specialized approach, applied only to carefully selected groups of patients. At the same time, there is growing interest in exploring the potential advantages of combining allo‐SCT with these new immunotherapeutic approaches, particularly in patients with high‐risk disease [15].

The advent of the first non‐covalent BTKi pirtobrutinib added a second major layer to this discussion. Pirtobrutinib provides meaningful activity with a substantially favorable toxicity profile therapy in patients failing a cBTKi. Reproducing pivotal trial results [2], real‐world data from an Italian series reported overall response rate of 47.6%, with CR in 23.8%, median progression‐free survival of 4.7 months, and median overall survival of 15.3 months; importantly, adverse events were predominantly grade 1–2, and only one patient discontinued treatment for toxicity [23]. Similar findings were reported by a European based series [24]. The Italian paper reported of nine patients proceeding to CAR‐T after pirtobrutinib, with encouraging short‐term outcomes after infusion. These findings support the view of pirtobrutinib not as a competitor to CAR‐T, but as a holding or “cool down” therapy, or post‐CAR‐T salvage option within a broader sequence‐based strategy. The NCCN patient guideline for MCL reflects the increasingly complex placement of BTK inhibitors, CAR T‐cells, and other novel agents in relapsed disease [25]. Although patient‐facing guidelines do not substitute for full professional algorithms, they illustrate how CAR‐T and newer targeted approaches are now embedded within a modern relapse strategy rather than reserved as isolated late‐line rescue tools [13, 14, 25]. Also last ESMO and EHA guidelines moved in this direction [13, 14].

## 5 Prioritizing the Use of CAR‐T

The rationale for moving CAR‐T earlier is now being tested prospectively. The CARMAN study has been designed as an international randomized phase II trial evaluating early treatment intensification with brexu‐cel in high‐risk MCL after abbreviated induction incorporating rituximab and ibrutinib, compared with standard‐of‐care induction and maintenance [26]. Studies like the CARMAN have the merit of shifting the field from retrospective inference to prospective testing of whether earlier cellular therapy can improve outcomes before cumulative treatment resistance occurs, as T‐cell dysfunction, or clinical deterioration reduce feasibility [19].

Taken together, the available evidence supports a more nuanced model for second‐line and early‐relapse patients (Figure 1). CAR T‐cells remains the treatment most strongly associated with deep and durable remissions in cBTKi‐exposed disease, and should continue to be prioritized in fit, eligible patients. However, the real‐world attrition between relapse and infusion, the toxicity burden after infusion, and the poor prognosis after CAR‐T failure argue strongly against a rigid linear algorithm. Instead, pirtobrutinib and possibly other novel agents with pending approval (i.e., nemtabrutinib, sonrotoclax, BTK‐degraders, etc.) should be incorporated as tools to maintain disease control, preserve CAR‐T eligibility, or provide salvage when CAR‐T is not feasible or has failed. In that sense, the key clinical challenge is not simply choosing between CAR‐T and pirtobrutinib but constructing the right sequence for the right patient at the right time.

**FIGURE 1:**
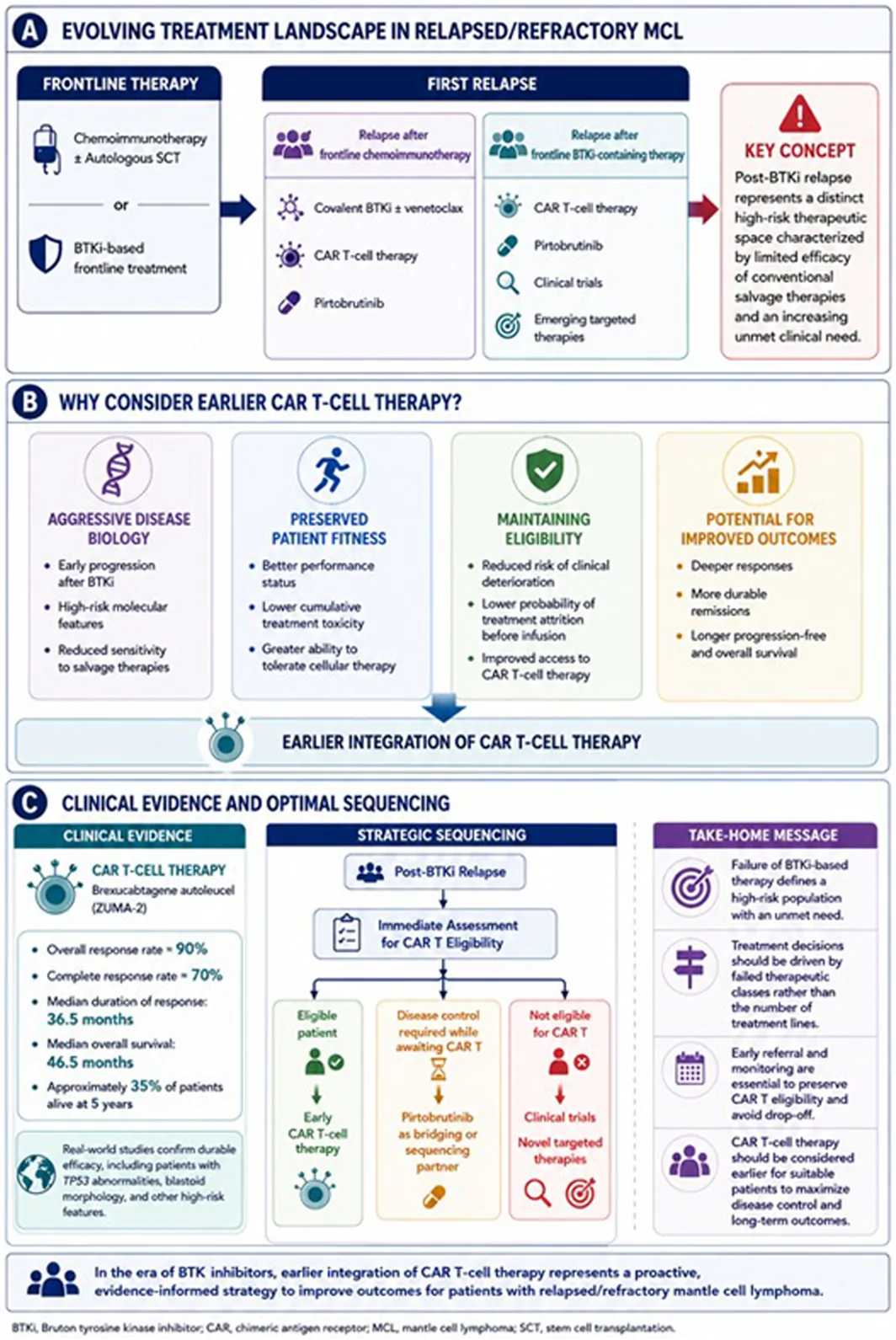
Strategic integration of CAR T‐cell therapy for mantle cell lymphoma in the era of BTK inhibitors.

Although the use of brexu‐cel in earlier treatment lines for BTKi‐naïve patients is expected to enhance clinical outcomes, most patients are likely to receive a BTKi prior to CAR T‐cell therapy within the current treatment paradigm as in the cohort one of the ZUMA 2 trial [5]. In clinical practice, the second‐generation BTKis, acalabrutinib and zanubrutinib, are increasingly preferred over the first‐generation agent ibrutinib because of their improved safety profile [4]. Nevertheless, current BTKi selection strategies do not consider the possible distinct impacts on the effectiveness of subsequent therapies. In a retrospective analysis of data and samples from ZUMA‐2, we observed associations between prior ibrutinib exposure and both efficacy/safety outcomes and effector‐polarized brexu‐cel phenotypes, compared with exposure to acalabrutinib [27].

Patients with R/R MCL who had received cBTKi upfront should be approached differently at relapse or progression. Patients who stop taking cBTKi due to side effects or time limited therapy are not necessarily refractory to the drug class, and can benefit of a rechallenge (with same or different drug) before proceeding to CAR‐T. Instead, for patients who progress during an active cBTKi therapy, a switch to entirely different classes of drug.

Finally, early CAR‐T in relapsed/refractory large B‐cell lymphoma is associated with lower hemato‐toxicity burden, and this aspect should be tested in MCL.

## 6 Conclusion

The clinical results that favor the use of upfront cBTKi in MCL are now mature and consolidated in both young and older patients [1, 2]. The use of chemo‐immunotherapy (as a class of drugs) and cBTKi (as second class of drugs) together in first line makes it clear that if a patient fails this line he has failed two classes of drugs. This theory would apply also to regimens based on doublets or triplets of targeted drugs, where immunotherapy (one class), and targeted options (second class) are delivered together (e.g., acalabrutinib, venetoclax, and rituximab or acalabrutinib, venetoclax, and obinutuzumab or zanubrutinib, obinutuzumab, and venetoclax). The results obtained with pirtobrutinib, which represents the only available option in Europe in this setting at the moment, are not as deep and durable as for CAR‐T, thus largely inferior.

Therefore, for the above‐mentioned reasons, in the interest of our patients with MCL, the scientific community should focus on the concept that it is not the number of lines that is important, but the classes of drugs that the patient has failed. This shifts in the interpretation of therapeutic lines would be desirable, otherwise we will have to resign to the use of less effective therapies in second line, in contrast with the real need of the patient himself.

## Author Contributions

**Carlo Visco:** conceptualization, writing – original draft, writing – review and editing, supervision. **Stefania Bramanti:** writing – original draft, writing – review and editing. **Paolo Corradini:** writing – original draft, writing – review and editing. **Enrico Derenzini:** writing – original draft, writing – review and editing. **Pier Luigi Zinzani:** conceptualization, writing – original draft, writing – review and editing, supervision.

## Funding

Medical writing support (editing and formatting) was provided by Mattioli Health and funding by unrestricted grant by Gilead Sciences S.r.l.

## Ethics Statement

The authors have nothing to report.

## Consent

The authors have nothing to report.

## Conflicts of Interest

Carlo Visco: Received honoraria from AbbVie, BMS, Astra Zeneca, Servier, Incyte, Roche, Pfizer, Novartis, Gentili, Johnson and Johnson, Kite‐Gilead, BeOne, Lilly, Kyowa Kirin and Sobi. Stefania Bramanti: no conflicts of interest to declare. Paolo Corradini: Received honoraria from AbbVie, Amgen, BeOne, BMS, Eli Lilly, Gilead/Kite, GSK, Incyte, Janssen, Novartis, Pfizer, Roche, Sanofi, SOBI, and Takeda. Enrico Derenzini: Received honoraria from Roche, Gilead, Lilly, Incyte, SOBI, Astra Zeneca. Abbvie, Gilead, Takeda, BMS, JandJ and Regeneron. Pier Luigi Zinzani: Received honoraria from BMS, Gilead Sciences, Roche, Kyowa Kirin, Sobi, Incyte, Novartis, Beigene, Janssen and AbbVie.

## Permission to Reproduce Material From Other Sources

The authors have nothing to report.

## Data Availability

Data sharing not applicable to this article as no datasets were generated or analysed during the current study.
